# Foundational Investigation on the Characterization of Porosity and Fiber Orientation Using XCT in Large-Scale Extrusion Additive Manufacturing

**DOI:** 10.3390/ma15062290

**Published:** 2022-03-20

**Authors:** Nevine Tagscherer, Thomas Schromm, Klaus Drechsler

**Affiliations:** 1 Chair of Carbon Composites, Department of Mechanical Engineering, TUM School of Engineering and Design, Technical University of Munich, Boltzmannstraße 15, 85748 Garching bei Muenchen, Germany; klaus.drechsler@tum.de; 2 BMW Group, New Technologies and China, Petuelring 130, 80788 Munich, Germany; 3 Chair of Non-Destructive Testing, Department of Mechanical Engineering, TUM School of Engineering and Design, Technical University of Munich, Franz-Langinger-Straße 10, 81245 Munich, Germany; thomas.schromm@bmw.de

**Keywords:** Additive Manufacturing (AM), digital manufacturing, void analysis, X-ray Computed Tomography (XCT), mechanical properties, fiber orientation, composites

## Abstract

The great potential of Extrusion Additive Manufacturing (EAM) for structural prototyping in the automotive industry is severely limited by the directional bias in the build direction. The layerwise fabrication leads to reduced mechanical properties at the layer-to-layer interface compared to the bulk of the strand. Especially for the often-used semi-crystalline thermoplastics, the mechanical properties strongly depend on the processing parameters, even more so if short fibers are used as fillers. Therefore, ideal processing windows in which the mechanical strength and modulus in the z-direction reach their maximum can be identified for these parameters, resulting in a reduced directional bias. The influence of the EAM processing parameters on mechanical strength has already been investigated, correlating strength with thermal conditions during printing. However, these considerations did not distinguish between the thermal effect on the polymer properties, the formation of voids and pores at the layer interface, and the resulting fiber orientation for different strand proportions. Therefore, in this study, the effect of different processing temperatures and layer heights on the pore size and distribution, as well as the fiber orientation in the different regions of the mesostructure was investigated using X-ray Computed Tomography (XCT).

## 1. Introduction

Additive Manufacturing (AM) has become an effective approach to produce small quantities or individualized products with high geometric flexibility [1,2]. By depositing material only where required, the part properties can be controlled locally [3] while generating minimal material waste, which is highly relevant for high-cost materials and from an environmental perspective. Progressing digitalization and automation of AM processes and the development of large-scale processes [4,5] offer a great potential for applications in the automotive industry [2]. Compared to laser- or powder-based systems, material-extrusion processes require only simple manufacturing facilities that at a large scale benefit from the use of injection molding granulate as the feedstock. Pelletized polymeric feedstock greatly reduces the material cost while achieving much higher deposition rates [4].

The major deficit of Extrusion Additive Manufacturing (EAM) compared to conventional polymeric manufacturing techniques is the directional bias resulting from the layer-by-layer deposition [4,6,7,8]. Due to incomplete fusion bonding across the interlayer interface, the molecular entanglement is inferior to the bulk of the strand, resulting in increased porosity along the weld line. Both destructive and non-destructive methods have confirmed this anisotropy with reduced mechanical strength and an increased number and volume of voids in the build direction [8]. As expected [9,10], previous flexural and tensile testing of EAM specimens showed a high mechanical anisotropy due to reduced mechanical properties along the layer interfaces, requiring further investigation and optimization [11,12].

The choice of material is decisive for the effectiveness and applicability in industrial structural manufacturing. The use of thermoplastics instead of thermosets benefits from re-meltability due to lack of crosslinks and yields better mechanical and chemical characteristics, recyclability, reparability, lower costs, and shorter cycle times [13,14,15]. However, increased processing temperatures and pressures are required to overcome their high viscosity and to achieve active flow in EAM for deposition [16,17]. Semi-crystalline thermoplastics in particular are readily available for other manufacturing processes, such as injection molding, and have already been qualified for automotive and transport applications [18]. Due to their complex semi-crystalline structure and the strong dependence of crystallization on the thermal conditions during processing, the final part properties and the mechanical strength of the weld line strongly depend on the processing parameters selected [15,18,19]. Previous investigations correlated this high dependency of the resulting mechanical properties to the characteristic properties of the polymer [11].

Polyamide 6 (nylon) is a common engineering polymer with good heat and stress resistance [20], used as base polymer in automotive injection molding. For prototyping similar parts, the same feedstock polymer, but with increased fiber reinforcement content was chosen. The use of short carbon fibers as fillers is a measure to improve the base polymer properties [10,21,22]. The increase in filler content compared to injection molding contributed to increasing the part stiffness and to reducing warping during manufacturing [23,24,25]. This improved processability and the elimination of the required envelope heating in the assembly space enabled the further development of effective large-scale EAM processes. Based on this, an automotive-grade re-meltable semi-crystalline polymer was selected as feedstock for this study. Due to its established application in injection molding, Akromid B3 ICF40 by AKRO-PLASTIC GmbH as polyamide 6 reinforced with 40 wt% chopped and recycled carbon fibers was chosen.

**Figure 1:**
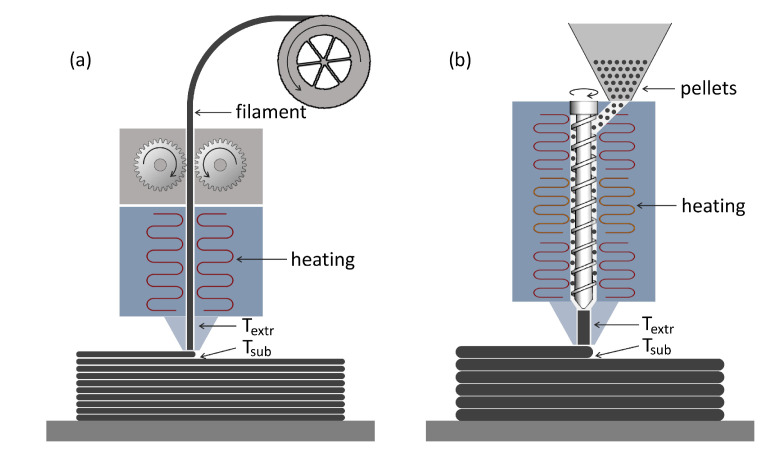
Concept comparison between (**a**) Fused Filament Fabrication (FFF) and (**b**) large-scale Extrusion Additive Manufacturing (EAM).

One of these novel EAM processes is the BMW Group’s Freeform Extrusion Additive Manufacturing (FEAM) approach developed at the BMW Landshut plant. The objective of the FEAM system was cost-effective prototyping of injection molded parts in the early stages of the product development process and the manufacturing of structural parts for concept vehicles. The system works similar to Fused Filament Fabrication (FFF), in terms of depositing molten polymer beads in a layerwise manner. However, to increase the throughput and effectiveness of the process and to reduce the material costs, the filament was replaced by pelletized feedstock, as commonly used in injection molding. Accordingly, the filament feed system was replaced by a single screw extruder that melts and conveys the polymer right before deposition. Figure 1 presents the comparison of the common FFF process and the EAM process employed in this study. The robot-based large-scale EAM system in an unheated printing envelope with an unheated print bed is natively equipped with a 2.0 mm nozzle and represents a good compromise between fast manufacturing times, high material throughput, and small wall thicknesses, which are similar to injection molding. The use of a 2.0 mm nozzle led to layer heights between 0.8 mm and 1.0 mm. Below 0.8 mm, the material was not deposited homogeneously and led to large defects, while layer heights above 1.0 mm led to insufficient layer adhesion and inhomogeneous print results, as well as increased surface roughness [9]. While the majority of the intended parts required single-strand manufacturing, individual mechanically loaded parts required multi-strand walls. Therefore, we differentiated between thin-walled structures, in which the wall thickness is identical to the width of a strand, and tick-walled or volumetric structures, in which adjacent strands are fused together to create greater wall thicknesses, as in to common 3D-printing processes.

Most EAM analyses of finished parts or processing parameters focus exclusively on the mechanical properties. One problem with this is the neglecting of porosity, although studies have shown that processing parameters strongly influence the shape, size, and spatial distribution of micropores and macropores [22], which in turn affect the properties of the mesostructure and the finished part [26]. Furthermore, the parabolic velocity profile of the melt and varying shear conditions within the extrusion unit significantly affect the fiber orientation during deposition. With fibers expected to align in the flow direction, the induced fiber alignment greatly influences the final parts’ thermal and mechanical properties. If the influence of processing parameters on porosity, fiber orientation, and mechanical properties is known, the print strategy can be optimized to tailor part properties according to specific load cases [3].

There are several approaches to fiber and porosity analysis. The most common method is the examination of photomicrographs through optical or incident light microscopy [3,27]. Although these microscopes are readily available and the specimens are easy to manufacture, these micrographs present only a two-dimensional (2D) section of the entire part and make it increasingly difficult to determine pore volumes, fiber orientations, and the overall distribution of the characteristic mesostructural features. Scanning Electron Microscopy (SEM) builds on this by increasing the depth of focus to a larger range, enabling the visualization and analysis of three-dimensional (3D) mappings of the strands’ outer surface or fracture surface. This enables the evaluation of fiber distribution and fiber–matrix adhesion at the surface [28,29]. However, both approaches only consider a superficial view of the outer surface of a part and are incapable of fully investigating the inner mesostructure of an intact strand or surface. Therefore, X-ray Computed Tomography (XCT) was chosen as an effective and non-destructive volumetric measurement tool with the capability for 3D investigations of microscopic structural details [27,30]. Although XCT scans are limited by the material contrast that can be achieved to detect the same characteristic features as high-resolution optical microscopy [31], the initial scans provided enough detail to determine pores, fibers, as well as their size and distribution.

The predicament of previous XCT analyses for AM [8,30] was the focus on high-resolution or high-density processes with little consideration of polymer extrusion-based characterization. Especially, the use of granulated feedstock material, as well as unheated build volumes in large-scale EAM approaches require a thorough understanding of the material behavior and the influence of the processing parameters on the porosity and fiber orientation of the final product. It was hypothesized that, due to the use of semi-crystalline thermoplastics, the thermal conditions during manufacturing in combination with the forcing of the flow by different layer heights greatly impact the resulting interlayer and intralayer interfaces. For this, the influence of the interface temperature and layer height on the distribution of voids, pores, and fibers had to be determined for both single-strand and multi-strand parts.

## 2. Materials and Methods

Since a full factorial Design of Experiments (DoE) would have been beyond the scope of this work, a fractional factorial screening was chosen. The aim was to analyze the fundamental influence of processing temperatures, layer heights, and the air gap on fiber orientation, pore content, and pore distribution, as well as the overall quality of the interface. Thorough material investigations and mechanical testing [11] had previously identified an ideal processing window for the substrate temperature at the deposition of the subsequent layer. This processing window ranged from the melt temperature to the onset of crystallization, with greatly reduced mechanical properties below the lower limit at 166 °C. The substrate temperature was chosen as a control parameter, since it could be monitored continuously without any process interference, while being the decisive factor for the resulting interface temperature.

Previous investigations on the major influencing parameters for optimized fusion bonding determined that the greatest influence for high bond strength and elastic moduli was the interface temperature that results from the contact of the already-deposited substrate and the newly deposited extrudate [12]. While the layer height was expected to have no effect on the strength per unit area, a minimal deviation was found with lower layer heights, resulting in a slightly reduced tensile strength and modulus. Since the extrusion velocity of the bead is constantly coupled to the traversing velocity to avoid pulling or pushing of the strand, the volume of deposited polymer in FEAM is non-varying and fully independent of the chosen layer height, resulting in different bead proportions. The authors suspected that different bead proportions and greater pressure of the nozzle on the bead due to a reduced layer height may lead to different fiber alignments and porosity distributions along the interface, which in turn could explain the determined minor reduction in strength for lower layer heights. Therefore, the DoE was constructed to investigate the influence of the highly important substrate temperature, as well as, at a small scale, the impact of varying layer heights as the explanation for the changes in tensile strength per unit area.

All samples were manufactured at an unheated envelope and print bed temperature of 23 °C. Previous investigations found the cooling behavior to depend solely on the layer time resulting from the path length, traversing velocity, and potential wait times. Therefore, no consideration of different traversing speeds was required, and a variation in the substrate temperature could be achieved by different wait times [12]. This allowed keeping the part size and resulting heat chimney, as well as the traversing velocity and screw speed constant. By this, the authors ensured that the processing history and state of the extrudate were identical for all specimen setups, eliminating unnecessary disruptive factors. The extrudate temperature $T_{extr}$ could therefore be kept constant according to the manufacturer’s recommendations at 280 °C. The layer length of the single-walled samples was set to 1.40 mm, the traversing and thus extrusion velocity to 0.160 m/s, and the screw speed to 72.04 rpm. Through the use of a wait time $t_{w}$ between 0.1 s and 18.0 s at the end of every layer, substrate temperatures between 217 °C and 130 °C were obtained. The layer length of the triple-walled samples was 1.80 m, with the polymer deposited at a velocity of 0.160 m/s and the corresponding screw speed of 72.04 rpm, identical to the single-walled samples.

Due to the limited number of scans possible, samples had to be selected carefully. To investigate the influence of the temperature, 10 mm × 10 mm samples were taken from parts manufactured at substrate temperatures of 130 °C, 150 °C, 170 °C, 200 °C, and 217 °C, with 130 °C and 150 °C falling outside of the suggested ideal processing window, resulting in reduced mechanical properties. With a layer height of 1.0 mm as the default parameter, the influence of the layer height was investigated only for the lower and upper limit of the ideal processing window, for the specimens at 170 °C and 217 °C for additional samples of a 0.8 mm layer height. No additional pressure or compaction were applied by the system during printing, and no post-treatment was applied to the samples.

**Figure 2:**
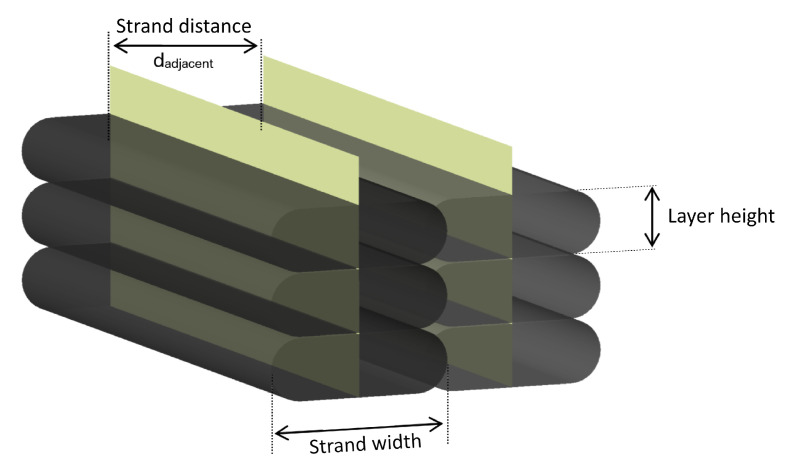
Schematic representation of a multi-walled sample at a strand distance with a negative air gap to reduce the macro-porosity between strands.

Multi-strand specimens were also investigated for components with wall thicknesses greater than a single strand. Since the interlayer behavior was expected to be analogous to the single-walled specimens, the strand distance between adjacent strands in the single layers was chosen as a variable parameter at constant layer heights of 1.0 mm and a substrate temperature of 200 °C. As presented in Figure 2, the macro-porosity depends on the air gap resulting from the selected strand distance. This air gap should be negative in order for a minimum amount of pores to remain within the part [3,17,32,33].

From a purely mathematical point of view, the cross-sectional area of the individual strands determined by reflected light microscopy would result in a completely filled bulk part at a strand spacing of 2.75 mm and a strand width of 3.00 mm. Therefore, the maximum strand distance was chosen at 2.8 mm. The minimum strand spacing at which homogeneous deposition without material accumulation and defects was achieved was 2.5 mm. The objective of all XCT scans was to identify all closed pores that represent fully enclosed voids as sub-surface porosity [26] and to identify the fiber orientation within the different regions of the part. The samples were scanned via XCT and were imported into the analysis software (VGStudio 3.0) for fiber and porosity analysis, based on the manually defined Region Of Interest (ROI) of each sample.

Table 1 presents the selected XCT scan parameters. All scans were performed on a Waygate v|tome|x M 240 d system with no filters applied. The tube acceleration voltage had to be increased by 10 kV for the multi-walled samples because they represented larger geometries (~6 mm of additional material) along the X-ray beam direction. This resulted in more radiation absorption and thus less intensity information on the detector from which to reconstruct a high-contrast volume.

## 3. Results and Discussion

**Figure 3:**
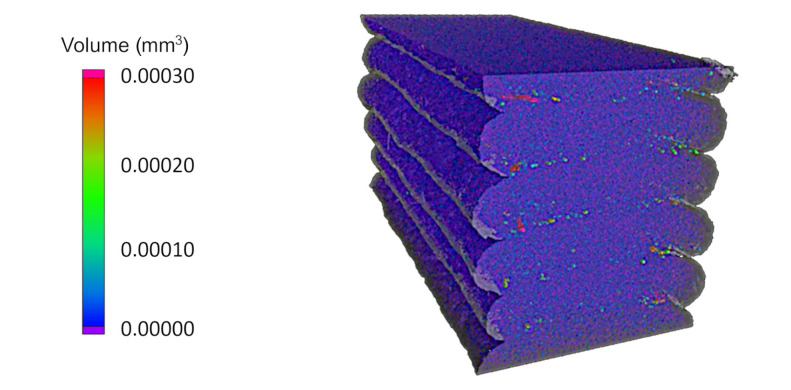
Three-dimensional view of the pores of a single-strand specimen at a substrate temperature of 170 °C and a layer height of 0.8 mm.

In a first step, the pores of the different ROIs of the single-strand scans were investigated in terms of shape, size, and distribution. The XCT analysis provided the option for a three-dimensional consideration of the individual specimens, as presented in Figure 3. However, the anisotropy of the analyzed composite AM parts is generally considered within the individual planes along the x-, y-, and z-axes. AS opposed to two-dimensional micrographs, the XCT scans were able to create an unlimited number of two-dimensional mappings and to determine individual pore volumes and characteristics. The further analysis was therefore focused on three-dimensional considerations aligned with the respective manufacturing planes for frontal, side, and top observations.

**Figure 4:**
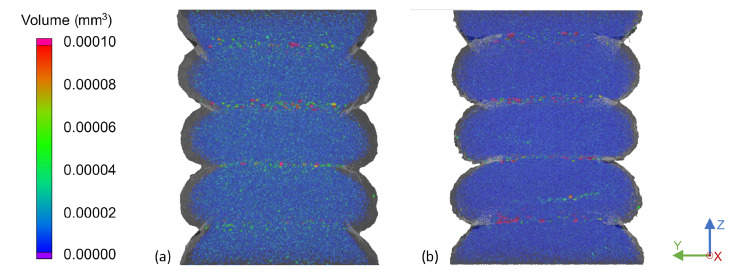
Front view of the pores of a single-strand specimen at a substrate temperature of (**a**) 200 °C and (**b**) 130 °C and a layer height of 1.0 mm.

Overall, a higher interlayer porosity was determined compared to the bulk of the strand, considering both the size and amount of pores. Figure 4 presents the front view of the 200 °C and 130 °C samples with a layer height of 1.0 mm. Smaller pores were located in the strands’ bulk structure, while larger pores resided at the layer interface. Interestingly, no pores were found in the boundary regions of the strands on any of the free surfaces in all of the setups. It was suggested that this was due to the air, which was induced during the processing of the material and in the extrusion unit, diffusing through the outer regions of the strand prior to surface curing.

The influence of different substrate temperatures at deposition becomes apparent in the comparison in Figure 4. Higher temperatures consistently resulted in flat layer top surfaces and layer interfaces, while temperatures below 170 °C obtained slightly convex strands and thus arched interfaces. This may be due to the substrate being significantly colder at deposition with a solidified outer layer, compared to a nearly molten state of lower viscosity at higher substrate temperatures. In this case, the freshly deposited extrudate pressed down on the substrate, flattening the surface under pressure and heat. This agreed well with the results of prior tensile tests, in which the mechanical strength and tensile modulus were strongly reduced for temperatures below 170 °C. The results of this work supported the assumption that this reduction in strength was not solely due to reduced fusion bonding ability, but also to an increased number and volume of pores at the interface. Across all samples, the influence of the temperature led to a few small- and medium-sized pores for high substrate temperatures and to more and larger pores at lower substrate temperatures. In addition it was noticeable that the rounded sides of the strands of Sample (b) in Figure 4 sagged downwards more than for the hotter Sample (a).

**Figure 5:**
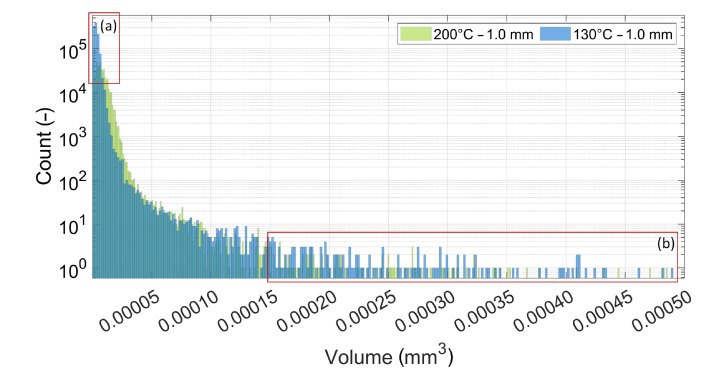
Quantitative analysis of the pore count for different pore volumes for the single-strand specimen at a substrate temperature of 200 °C and 130 °C and a layer height of 1.0 mm.

For both samples presented in Figure 4, a quantitative analysis of the pore amount and size was conducted as presented in Figure 5. The histogram shows that the colder sample at 130 °C contained significantly more small pores than the 200 °C sample, as highlighted by Region (a). However, the more substantial result was the increased amount of large pores presented in Region (b) of the histogram. These pores in Region (b) correspond to the large pores visible in Figure 4 at the layer interface, which may, in addition to reduced fusion bonding, be a cause for the reduced mechanical strength across the interface at lower substrate temperatures.

**Figure 6:**
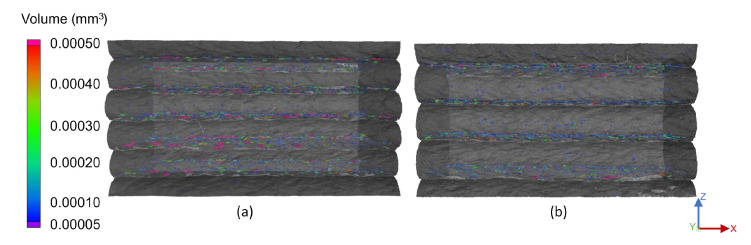
Side view of the porosity of two single-strand specimens at a substrate temperature of 170 °C and a layer height of (**a**) 0.8 mm and (**b**) 1.0 mm.

The influence of the layer height was investigated using four experimental setups, covering the ideal minimum and maximum substrate temperatures of 170 °C and 217 °C and the minimum and maximum layer heights of 0.8 mm and 1.0 mm. Figure 6 depicts the side views of both samples at a 170 °C substrate temperature with pores larger than 0.00005 mm${}^{3}$. The 0.8 mm layer height sample on the left shows larger pores at the interface, especially towards the free edges. The same effect was identified for the 217 °C samples, but to a lesser extent. In addition, a layer height of 1.0 mm led to more spherical pores, while lower layer heights yielded more irregular pores.

**Figure 7:**
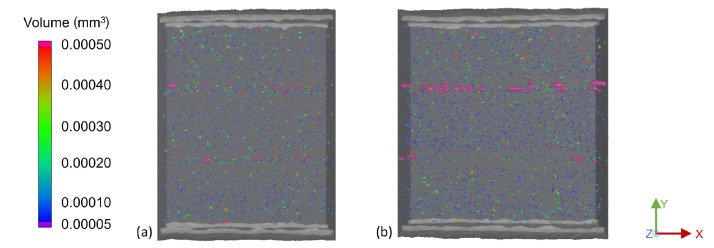
Top view of the porosity of two triple-strand specimens at a substrate temperature of 200 °C and a layer height of 1.0 mm with strand distances of (**a**) 2.5 mm and (**b**) 2.8 mm.

The increased interlayer porosity observed in the single-strand samples compared to the mass of the strand was confirmed by the multi-strand samples. With the objective of identifying the ideal strand distance by considering the air gap between adjacent strands, the macroporosity was evaluated. Figure 7 depicts the top views of the 2.5 mm and 2.8 mm strand distance samples. It is noticeable that the 2.8 mm sample on the right has significantly more larger, elongated pores between the strands, compared to the 2.5 mm sample on the left. In addition, a greater number of small- and medium-sized pores was present in both the bulk of the strand and the layer interfaces. The 2.6 mm samples contained less large pores than the 2.8 mm samples, but more large pores than for the 2.5 mm strand distance. Due to the increasing microporosity and macroporosity with increasing strand distance, it is advised to use the smallest strand distance feasible, in this case 2.5 mm.

**Figure 8:**
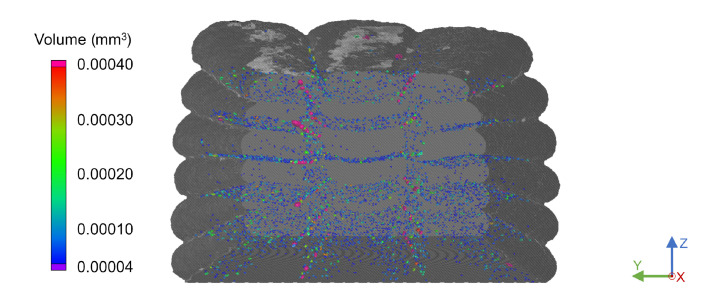
Front view of the porosity of a triple-strand specimen at a substrate temperature of 200 °C and a layer height of 1.0 mm with a strand distance of 2.5 mm.

Notably, the intralayer porosity between adjacent strands was significantly lower than the interlayer porosity across all multi-strand scans. This may be due to the higher substrate surface temperature when an adjacent strand is deposited compared to when the subsequent layer is extruded. With this leading to a higher interfacial temperature, chain mobility increased, resulting in greater adhesion between adjacent strands. As presented in Figure 8, the top-most layers appeared to be more convex than the lower layers. This was evident for all triple-strand samples, as they were taken from the top section of the components and not from the middle of the parts, as was the case with the single-walled specimens. While this indicated that the final strand shape was determined by the deposition of subsequent layers, no significant impact on the interlayer or intralayer porosity was evident.

**Figure 9:**
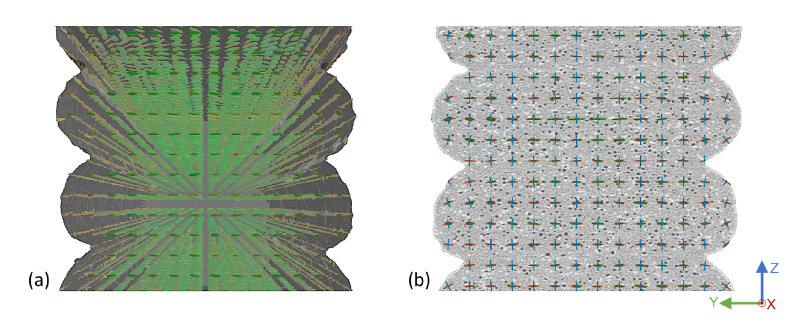
Front view of the fiber orientation tensors of a single-strand specimen at a substrate temperature of 200 °C and a layer height of 1.0 mm in (**a**) a 3D representation and (**b**) a 2D sectional image.

Following the porosity analysis, the effect of varying substrate temperature and layer height on the fiber orientation was investigated. For this purpose, each ROI was divided into a 3D mesh, with the fiber orientation tensors determined for each mesh element. All samples resulted in fiber orientation tensors predominantly parallel to the print basis, as depicted in Figure 9. While this was consistently the case in the bulk of the strand, the rounded edges of each strand showed a slight rotation about the x-axis.

**Figure 10:**
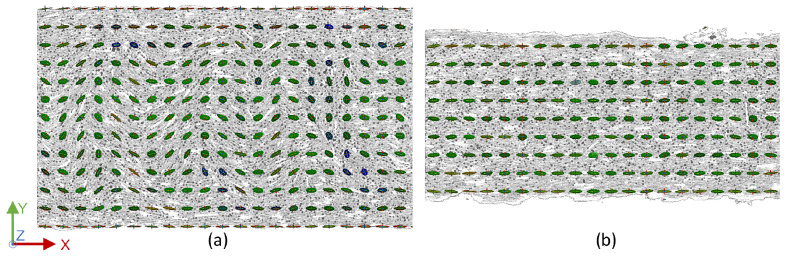
Top view of the fiber orientation tensors of a single-strand specimen at a substrate temperature of 130 °C at (**a**) the center of a strand and (**b**) at the interface between two strands.

Contrary to the expectation, the fibers did not fully align in the print direction. As depicted in Figure 10 on the left, only the sides of the strand aligned directly in the x-direction in the center of the strand, while the alignment in the bulk of the strand followed a wave-like profile. At the layer-to-layer interface on the right, all fiber orientation tensors aligned along the x-axis. It was presumed that this was due to the parabolic velocity profile of the melt and the varying shear conditions [34], resulting in highly oriented boundary regions of the strand with a slightly less oriented core. The fact that the fibers in the center regions were not aligned directly in the x-direction, but remained in the x–y-plane was attributed to the pressure and forced flow resulting from the nozzle pressing down on the newly deposited material. Only minor deflections in the z-direction were visible for lower temperatures, while identically aligned fibers were evident in the multi-strand samples. In contrast to the results of the porosity analysis, varying the substrate temperature and layer height showed no significant effect on fiber orientation. The triple-strand scans showed the identical behavior to the single-strand samples, as well as higher fiber orientation between adjacent strands compared to the bulk structures, confirming a more highly aligned boundary region in each individual strand, depending on the deposition itself.

It should be mentioned that the XCT equipment was not specifically calibrated for the measurements. By this, the absolute dimensions of the pores and overall parts may deviate slightly from reality. However, the ratio of pores to the overall structure will be correct. The comparability between the different measurements was achieved through identical scanning parameters with no individual adjustments to the calibration setup. Due to the limited number of possible scans, the number of setups and parts was limited. Therefore, the trends obtained can only be indicative and should be further investigated. The authors suggest a full factorial approach of XCT scans in combination with thorough mechanical testing. While the presented study determined a high potential of XCT for the fiber and porosity analysis, this would allow the concrete identification of ideal processing parameters for structurally loaded parts.

## 4. Conclusions

The objective of this investigation was to assess the suitability of XCT scans for the analysis of fiber-reinforced additive manufacturing parts, as well as to gain a basic understanding of the relevant processing parameters. Ultimately, the investigation of the fundamental influences of substrate temperature and layer height on porosity and fiber orientation in EAM samples provided new basic insights for the optimization of processing parameters and aids as the initial basis for further investigations.

In addition to limited thermally driven fusion bonding, the directional bias for lower processing temperatures was also attributed to the increased number and size of pores along the layer-to-layer interface. Substrate temperature also had an effect on the shape and proportion of the strands, with higher temperatures resulting in flatter interfaces and less deflection of the rounded strand sides. Increasing the layer height directly reduced the number and size of pores at the interface. This coincided well with the aim for short manufacturing times, since increasing the layer height reduced the number of layers required and thus the printing time. Unexpectedly, almost no pores were present near the free surfaces of the strands in both the single-strand and triple-strand samples, resulting in a pore-free boundary region. Due to lower macroporosity and only small pores at the interface, a strand distance of 2.5 mm was determined to be best suited for manufacturing with a 2.0 mm nozzle. The fiber orientation tensors evinced that the fibers in the bulk of the strands were not fully aligned, but were oriented in a wave profile due to the forced flow in the x–y plane during deposition.

While providing an initial approach to foster the understanding of thermal and dimensional processing parameters, this study presented X-ray computed tomography as a suitable method for the analysis of the fiber-reinforced EAM samples and their mesostructure. The first indications of increased porosity for lower layer heights and wider strand proportions were in line with the slightly reduced mechanical properties from previous studies [12]. Furthermore, the results indicated that the substrate temperature influenced both the shape retention and homogeneity of the build-up, as well as the porosity within the samples. The authors suggest further full factorial investigations of a larger set of processing parameters to find the optimum process setup for geometrically accurate, loaded structural parts.

## Figures and Tables

**Table 1:** XCT scan parameters for both the single- and multi-walled samples.

Sample	FDD	FOD	M	Voxel Size	Voltage	Current	Exposure Time
single strand	815.83 mm	32.19 mm	25.34	7.9 μm	40 kV	110 μA	750 ms
multi-strand					50 kV		

## Data Availability

The data presented in this study are available upon request from the corresponding author.

## Abbreviations

The following abbreviations are used in this manuscript:

2D	Two-Dimensional
3D	Three-Dimensional
AM	Additive Manufacturing
DoE	Design of Experiments
EAM	Extrusion Additive Manufacturing
FEAM	Freeform Extrusion Additive Manufacturing
FFF	Fused Filament Fabrication
FDD	Focus Detector Distance
FOD	Focus Object Distance
ROI	Region of Interest
XCT	X-ray Computed Tomography
